# Structurally Governed Cell Mechanotransduction through Multiscale Modeling

**DOI:** 10.1038/srep08622

**Published:** 2015-02-27

**Authors:** John Kang, Kathleen M. Puskar, Allen J. Ehrlicher, Philip R. LeDuc, Russell S. Schwartz

**Affiliations:** 1 Lane Center for Computational Biology, Carnegie Mellon University, Pittsburgh, PA 15213, USA; 2 Dept. of Mechanical Engineering Technology, Point Park University, Pittsburgh, PA 15222, USA; 3 Dept. of Bioengineering, McGill University, Montreal, Quebec H3A 0C3, Canada; 4 Dept. of Mechanical Engineering, Carnegie Mellon University, Pittsburgh, PA 15213, USA; 5 Dept. of Biological Sciences, Carnegie Mellon University, Pittsburgh, PA 15213, USA

## Abstract

Mechanotransduction has been divided into mechanotransmission, mechanosensing, and mechanoresponse, although how a cell performs all three functions using the same set of structural components is still highly debated. Here, we bridge the gap between emerging molecular and systems-level understandings of mechanotransduction through a multiscale model linking these three phases. Our model incorporates a discrete network of actin filaments and associated proteins that responds to stretching through geometric relaxation. We assess three potential activating mechanisms at mechanosensitive crosslinks as inputs to a mixture model of molecular release and benchmark each using experimental data of mechanically-induced Rho GTPase FilGAP release from actin-filamin crosslinks. Our results suggest that filamin-FilGAP mechanotransduction response is best explained by a bandpass mechanism favoring release when crosslinking angles fall outside of a specific range. Our model further investigates the difference between ordered versus disordered networks and finds that a more disordered actin network may allow a cell to more finely tune control of molecular release enabling a more robust response.

While the understanding of biological responses to extracellular matrix mechanical stimuli in diverse areas such as stem cell fate^1^, cancer metastasis^2^ and neovascularization^3^ has grown substantially, many details about the precise mechanisms by which intracellular molecules are able to assess forces are only beginning to be better understood. Nevertheless, a basic picture of cytoskeleton force transmission has emerged that connects interior and exterior cellular mechanics. Extracellular mechanical forces are transmitted from outside the cell via transmembrane integrins to the cytoskeleton through focal adhesion complexes^4^,^5^. Actin cytoskeleton dynamics are further heavily regulated by the Rho family of GTPases, including Rac1 (Rac)^6^. The critical interface between force transmission and sensing has recently been explored via force-uncovered exposure of cryptic sites^7^,^8^ and catch bonds^9^,^10^,^11^. Filamin, a ubiquitous actin crosslinker, is a natural homodimer^12^ and has been identified as a key mechanotransductive protein with over 90 partners^13^,^14^. Filamin's carboxy-terminal rod 2 domain has a compact structure yet can undergo conformational changes at 10 pN or less^15^ whereas filamin as a whole unfolds at much higher forces^16^, strongly implicating rod 2 as having mechanosensitive function. The rod 2 domain is especially interesting because it is both promiscuous—binding several key mechanotransductive proteins such as FilGAP^17^,^18^, Rho^19^, Rac^19^, Cdc42^19^, ROCK^20^, ICAM-1^21^, and integrin^22^,^23^—and because it directly borders the self-association hinge domain which flexes during mechanical stimulation^24^. Reconstituted studies show that mechanically stretching actin filament networks crosslinked by filamin A (FLNa) influences the release rate of FLNa-bound FilGAP^25^, an inhibitor of Rac^17^, suggesting a specific mechanoregulatory role for FLNa. The exact atomic structure for the FLNa rod 2-FilGAP interaction is unknown^13^ and high resolution structures of full-length filamin characterizing relevant crosslinking angles are lacking due its large, flexible nature and complex scaffolding^14^,^26^. In the absence of these structural details, it is not possible to analytically predict the quantitative strain-dependent kinetics of FLNa binding partners.

Here, we focus on the mechanotransductive response, using FLNa as a model system, by building a multiscale structural model to examine integrated mechanotransmission, mechanosensing and mechanoresponse (Fig. 1a). For full details, please refer to the Methods. Briefly, we first incorporate mechanotransmission through simulating stretch across a discrete network of actin filaments and associated binding proteins (Supplementary Fig. S1). These forces cause conformational changes at the crosslinking complexes. Previous studies on FLNa-FilGAP interactions have postulated that the homodimer FilGAP contains binding sites that interact with corresponding dimeric sites on FLNa whereby strain-induced separation of the FLNa dimers induces decreased avidity to FilGAP^24^,^25^. However, the exact determination of how this occurs is not known. Here, we hypothesize and test three geometric mechanosensing mechanisms for determination of this protein activation (Fig. 1b). Furthermore, we simulate the release of signaling factors from mechanosensitive crosslinkers using a time-dependent mechanoresponse mixture model that we parameterize with our experimental data^25^. Finally we examine the effects of network order versus disorder on these mechanotransduction responses.

We base our integrated mechanotransduction model on an architectural foundation to simulate the mechanotransmission of force across cytoskeletal structural elements by initially creating a discrete-element architectural model of square-grid actin filaments crosslinked by FLNa (Fig. 1a). We specifically favor a simple, minimal model that abstracts the multitude of cytoskeletal binding proteins and other elements of cellular architecture in order to test whether the specific components modeled are sufficient to account for observed responses. We previously constructed a mechanical model of the cytoskeleton^27^ and experimentally investigated mechanotransduction in living cells related to cell structure^28^. We simulate stretch by displacing apical region peripheral nodes while fixing the basal region peripheral nodes and relax forces using Gauss Seidel iterations on the mobile internal crosslinks until nodal force equilibrium is reached^27^ (Supplementary Fig. S1). Next, we assess how force-induced changes in network morphology change crosslinking binding angle distribution (Supplementary Fig. S2). As stretch is progressively applied, the crosslinking angle distribution flattens whereas the distribution of positive change that the angles undergo (“delta angle”) shifts to larger angular changes; these same patterns were suggested by our previous experimental results^25^.

To connect force-induced cell architecture changes with protein conformation changes, we postulate three geometric mechanosensing mechanisms at individual molecular complex crosslinks based on known geometric molecular concepts^25^: absolute, delta, and bandpass angle thresholds (Fig. 1b and Supplementary Fig. S3). A crosslinking site releases a bound signaling factor at slow or fast rate depending on whether it is considered “below threshold” (inactive) or “above threshold” (active), respectively (Supplementary Movies S1–3). Once the threshold is passed at a specific angle, this angle's state is set as constitutively releasing FilGAP at a fast rate. Otherwise the angle is assumed to be releasing a slow rate. These threshold mechanisms were chosen due to the pivotal role that the rod 2-hinge region plays in molecular binding^12^,^22^ as well as the importance of force-uncovered cryptic sites in mechanotransduction^7^,^8^. We apply these thresholding models to examine how changes in crosslinking angles would affect their propensity for releasing signaling factors (Fig. 2) and to later identify the mechanosensing model most consistent with experimental data. Small changes in stretch input can yield a variety of angle distributions (Supplementary Fig. S2) and small model parameter changes can substantially shift the ratio of fast to slow release molecules at different stretch amounts (Fig. 2, far right column), providing controls by which a cell can support multiple mechanically-sensitive switches with distinct response patterns and allowing for high adaptability to differing mechanical environments.

To understand the temporal mechanoresponse of our geometric model, we implement a two-exponential mixture model (Equation 1):
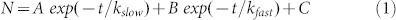
Equation (1) captures the slow and fast release of signaling molecules at crosslinker site populations below and above a given threshold, respectively. This overall signal *N* represents the number of signaling molecules remaining in a specific network configuration as a function of time. We determine *A* and *B* using our cell stretch simulations (Fig. 2) and we use Levenberg-Marquardt nonlinear regression to determine constants *k_slow_*, *k_fast_*, and *C* (Supplementary Table S1 and Supplementary Fig. S4). We first benchmark our mixture model to our experimental FLNa-FilGAP release data for unstretched and stretched networks to identify the release model best able to fit our experimental data^25^ (Supplementary Fig. S5). Our results suggest the best mechanosensing model is bandpass thresholding, yielding a minimum root mean square error (RMSE) of 0.100 at a 90 ± 7° band (Fig. 3c). Interestingly, this angle range is close to the natural orthogonal angle of FLNa-crosslinked actin filaments^12^,^18^. The best absolute threshold is 77° with RMSE 0.123 (Fig. 3a) and the best delta threshold is 0° with RMSE 0.262 (Fig. 3b). We perform a two-parameter search for the bandpass model to determine whether fitting both the width and the center of the band would improve the fit. We found that the new optimum of 92 ± 8° resulted in a negligible improvement in RMSE from 0.100 to 0.099 compared to the single parameter fit (Fig. 3d), suggesting that a single degree of freedom assuming an orthogonal center is sufficient to capture mechanotransductive behavior. The valley of low RMSE values observed in the two-parameter search suggests that cells potentially have significant flexibility for controlling bandpass release of different molecules.

In order to test the robustness of our model, we change the density of the network by increasing or decreasing the number of the elements (Supplementary Table S2). Our baseline network consisted of 60 peripheral nodes that when linked together resulted in 421 internal nodes and 960 filaments (Supplementary Fig. S1). The decreased-density network contains 40 peripheral nodes (33% decrease), 181 internal nodes (57% decrease) and 440 filaments (54% decrease). Decreasing the network density led to a best fitting absolute threshold of 82° (+5° from baseline), delta threshold 0° (same) and bandpass threshold 90 ± 6° (−1° width from baseline). The increased-density network contains 80 peripheral nodes (33% increase), 761 internal nodes (81% increase) and 1680 filaments (75% increase). Increasing the network density led to a best fitting absolute threshold of 77° (same), delta threshold 0° (same) and bandpass threshold 90 ± 9° (+2° width from baseline). These results suggest that while the specific threshold values showed some sensitivity to network parameters, the overall result, that bandpass threshold produces the best fit model, was robust to considerable network perturbation.

To further test the robustness of our model, we examine how the border geometry of our cytoskeletal network affects our fitting results. We change the peripheral node arrangement to a flat configuration in order to more closely mimic an experimental flat plate setup^25^ (Supplementary Fig. S6). Our results show that changing the peripheral node geometry from circular to flat did not substantially affect the relative number of thresholded angles but did affect their spatial location (Supplementary Fig. S6 vs. Fig. 2). Since the molecular release model does not take into account angle location but only their label as slow/fast releasing, there were no changes in the best fit thresholds and only minor RMSE differences: absolute threshold RMSE 0.112, delta threshold RMSE 0.259 and bandpass threshold RMSE 0.104 for the flat peripheral network compared to the RMSEs of 0.123, 0.262 and 0.100 for the corresponding thresholds in the original circular peripheral network. These results suggest that our model of FLNa-FilGAP release is robust not only to changes in parameter density but also to overall cytoskeletal network geometry.

Another important question in this field is the role that order in cellular structural geometry plays in shaping mechanotransduction. We approach this topic by building upon prior models of randomized actin network generation (Supplementary Fig. S7) to examine whether a strongly ordered square-grid actin cytoskeletal network, such as was described in Ehrlicher et al.^25^, yields distinct behaviors from a randomized disordered network that better reflects a biologically-relevant system. We randomize filament generation in our network (Fig. 4a) and examine model outputs using the previous methodology while keeping the square-grid optimized parameters as we assume FLNa-FilGAP behavior is independent of overall geometry (Supplementary Movies S4–6). Extension to random networks leads to a flatter distribution of angles due to more inherent noise in the system (Supplementary Fig. S8). Interestingly, the number of angles below threshold in a random network varies nearly linearly with the threshold (Fig. 4b), whereas in a square network this variation is much more dependent on the specific threshold values, with noticeable increases at lower values for delta and bandpass thresholding, and higher values for absolute thresholding (Fig. 4c). These results suggest that, while there is qualitative similarity between the models, a more ordered model would substantially understate the sensitivity and responsiveness of a disordered system. A more random network may allow the cell to more finely tune control of molecular release since small variation in thresholds can lead to predictable changes in the distribution of angles passing mechanosensitive thresholds. Together with the leeway in the center and width of bandpass thresholds, our results suggest that the cell has considerable flexibility to modulate architectural parameters in order to output a specific signaling response.

While we chose our parameters carefully in this study to emulate FilGAP release from FLNa, this is just one potential use of our model. Given the three isoforms of filamin (FLNa, FLNb, FLNc) and over 90 binding partners^13^—of which many bind within or in close proximity to the rod 2-hinge region—we postulate that different model parameterizations may apply to other mechanotransductive pathways that could be investigated in future work. One question our model may help solve is how the cell simultaneously controls the multiple mediators necessary in motility; having different crosslinker thresholds for different molecules would give the cell another control system to direct morphology changes. For example, the same external forces that weaken FLNa-FilGAP avidity have been shown to concurrently lead to stronger binding between FLNa and β-integrin β_7_^25^. Our model could be extended to investigate how two opposing phenomena can occur in the same cell by comparing different mechanosensing threshold mechanisms. With proper model parameterization and time-dependent experimental data, our methodology can be generalized to model either increased or decreased binding affinities to putative mechanotransductive binding partners near the FLNA hinge region such as Rho^19^, Rac^19^, Cdc42^19^, ROCK^20^, ICAM-1^21^. Another aspect our model could incorporate is probabilistic molecular release. Currently, a molecule's release rate is selected deterministically in a threshold-dependent manner, but it remains to be determined if incorporating stochastic release using factors such as binding energy could improve the quality of the fitting. The modularity of our model in separating mechanotransmission, mechanosensing and mechanoresponse allows incorporating improvements rather straightforward and will allow our field to explore a range of possible hypotheses for structural multiscale responses in mechanotransduction.

## Methods

### Mechanotransmission model for stretching the network

**Figure 1:**
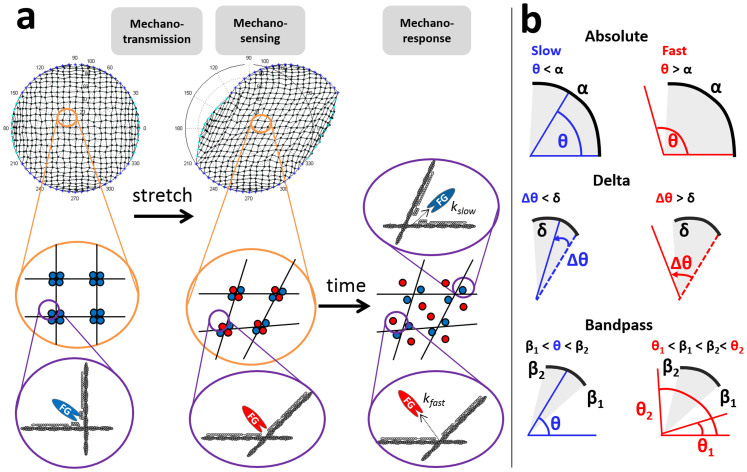
Structurally-governed multiscale cellular mechanotransduction. (a) Our approach bridges mechanotransmission, mechanosensing, and mechanoresponse through integrating structural and biochemical interactions. Mechanotransmission: we generate a square-grid discrete-filament network fixed at perimeter nodes. Crosslinking filaments form intersections containing four FLNa-FilGAP complexes, one at each angle (orange insets). We simulate mechanical stretching by displacing the perimeter nodes and iteratively relaxing network forces as previously described^27^. Mechanosensing: force transmission across the network alters crosslinking angles and, by extension, the binding affinities between FLNa and FilGAP^25^. (Orange insets) Here, we use an absolute threshold of 90° to categorize an angle as releasing FilGAP slowly (blue dots) or quickly (red dots). Mechanoresponse: we simulate time-dependent release at each angle. (Purple insets) Here, FilGAP (FG) molecules are released at rate *k_slow_* (blue) or *k_fast_* (red) depending on their mechanosensing threshold categorization. Actin filaments in dark grey, FLNa in light grey. Network parameters: 421 intersections, 60 peripheral nodes, and 960 filaments. (b) We test three thresholding models for determining slow (blue) or fast (red) FilGAP release. Absolute: fast if the angle exceeds α. Delta: fast if the angle increases relative to its starting value by at least δ. Bandpass: fast if the angle falls outside range [β1, β2].

Our default actin network model is represented as a discrete set of filaments in a two-dimensional circular solution space of prescribed radius. This network is considered fixed to an underlying substrate at pre-determined perimeter nodes, representative of focal adhesions fixing a cell on a substrate (Supplementary Fig. S1). Filaments representing actin filaments are formed by linking opposing focal adhesions on the periphery; these crosslinks can either be determinate in an ordered square-grid network (Fig. 1a) or a disordered random network (Fig. 4a). Intersections formed by crosslinked filaments represent molecular complexes of associated molecules (e.g., filamin A and FilGAP) and actin filaments at each of the four angles created by those intersecting filaments. This setup of a cytoskeleton consisting of interconnected nodes and filaments builds on our prior biomechanical model of actin networks^27^ (Supplementary Fig. S7).

The actin network simulation parameters used are 421 internal nodes, 60 peripheral nodes, and 960 filaments; altering the density of the network did not qualitatively affect our later results (Supplementary Table S2). We first implement a geometric network with ordered intersections to model the molecular system used in previous experimental studies and then extend to a more randomly-connected network intended to model a disordered actin cytoskeleton. For either variant, we can create a well-connected network of nodes and filaments that models the loose gel-like actin filament-filamin crosslinked networks found in specific areas of the cell, such as the cortex^14^.

Mechanical stretching is simulated by displacing predefined perimeter nodes on the apical region (i.e., an arc on the uppermost nodes) in a defined horizontal direction while fixing in place perimeter nodes on the basal region (i.e., an arc on the bottommost nodes). This action simulates a force displacing the apical layer of an epithelial cell while the basal focal adhesions are fixed to the basement membrane^25^. This movement of apical nodes creates imbalanced forces on filament-connected free nodes that are iteratively relaxed until force equilibrium is achieved using methodology described in our previous simulation work^27^. The magnitude of stretch is defined as the ratio of the horizontal displacement of the apical region nodes to the cell diameter, which is consistent with the experimental data^25^. We simulate stretch from 0–28% in 1% incremental steps.

The internal nodes act as force-movable hinges formed by intersecting filaments. These hinges at internal nodes model locations of the actin-binding protein filamin A, which is known to form relatively orthogonal angles in both truncated constructs^12^ and natively while crosslinking actin filaments^13^,^26^. As stretch is applied, the intersecting angle distribution transitions from a more peaked to a flatter distribution while remaining centered at 90°. The difference in the stretched angle relative to the non-stretched angle (“delta angle”) had a shift to larger values in distribution under the same stretch (Supplementary Fig. S2). These histograms reflect similar results to previous simulations of experimental molecular systems even though those simulations had different overall morphologies and boundary conditions^25^.

### Mechanosensing model for linking network architectural changes to filamin deformation

Our model implements mechanosensing by linking structural changes in the actin network to biochemistry through molecular deformation of actin crosslinkers leading to the release of previously-bound molecules. A system implicated in this approach is our model system of FilGAP release from FLNa^25^. Our interest in the mechanotransductive effect of crosslinks stems from previous studies hypothesizing the importance of external forces on protein structure geometry at the FLNa-FilGAP binding site^18^,^25^. Molecular dynamics studies on integrin—also implicated in FLNa-binding near the hinge—suggest that domain-domain hinge angles can be a mechanism for activation of mechanotransductive proteins^29^,^30^. In our model, we assume each internal node intersection holds a maximum of four FilGAP molecules—one at each angle—and that the rate of release of FilGAP is a function of the angular deformation of the binding site. Based on the intersection angles in the network, we assign either a slow or fast rate of release to the embedded molecule from the binding site. We test three different threshold methods for this assignment: absolute angle thresholds, delta angle thresholds, and bandpass angle thresholds (Fig. 1b and Supplementary Fig. S3). These thresholds represent simplified models of protein deformation whereby if an angle has not passed a threshold, the FLNa crosslink is considered to be in a conformational state of slowly releasing FilGAP at a rate *k_slow_*. Once an internal angle has passed a threshold, the crosslink is considered to be in a conformational state of quickly releasing FilGAP at a rate *k_fast_*. We initialize the model by assuming FilGAP is present at each binding site below threshold. For an absolute threshold of α, an angle θ is considered to be below or above threshold when θ < α or θ > α, respectively. For a delta threshold of δ, a positive change in angle Δθ is considered to be below or above threshold when Δθ < δ or Δ θ > δ, respectively. For a bandpass threshold of [β_1_, β_2_], an angle θ is considered to be below threshold when β_1_ < θ < β_2_ and above threshold when θ < β_1_ or θ > β_2_. Once an angle is above threshold, we assume that it is constitutively activated to release at rate *k_fast_*. Using these thresholding models, we can identify and simulate subsets of fast-releasing and slow-releasing binding sites over time as mechanical stimulus is applied to the network.

### Mechanoresponse model for linking model of filamin deformation to molecular release

We next apply our mechanosensing filamin deformation model to a mechanoresponse model. Our aim here was to find the optimal mechanoresponse model that can best recapitulate our previous time-dependent fluorescence decay measurements of FilGAP concentrations as functions of time at stretch values of 0% and 28%^25^.

As a preliminary step in order to determine the optimal mechanoresponse model and rate constants, we perform curve fitting to the experimental data via the “nlinfit” MATLAB function, which uses Levenberg-Marquardt nonlinear least squares algorithm for nonlinear regression. We test four candidate mechanoresponse models of the general form for stretched exponential decay *N = A exp(−t/k) + C* that had been previously suggested to model stretched vs. non-stretched FilGAP fluorescence signal^25^. As an additional check, to determine whether the results are affected by explicitly modeling diffusion effects, we compare raw experimental data with data corrected by subtracting 0.5e^−*t*/0.15 s^ from the normalized raw data to generate diffusion-corrected normalized data consistent with previous methods^25^. The different objective functions tested and rationale behind them are detailed in Supplementary Table S1 and the plots with goodness-of-fit results are show in Supplementary Figure S4. One of the mechanoresponse models tested was the original Ehrlicher et al., Nature 2011 model that fit to diffusion-corrected data resulting in decay constants *k_fast_* = 0.5673 s and *k_slow_* = 3.6428 s, consistent with previously published constants^25^.

Our model selection results suggest that a mixture model of two exponential decays, where the decay constants remained consistent for unstretched and stretched networks, improved fitting compared to the Ehrlicher et al., Nature 2011 model. Additionally, our mixture model had lower complexity (number of fitted parameters/degrees of freedom) as a byproduct of using a common set of parameters *k_slow_, k_fast_* and *C* for both unstretched and stretched networks. We did not see a significant effect difference from modeling diffusion and thus we use the raw data, which does not explicitly correct for diffusion, for the rest of this paper.

**Figure 2:**
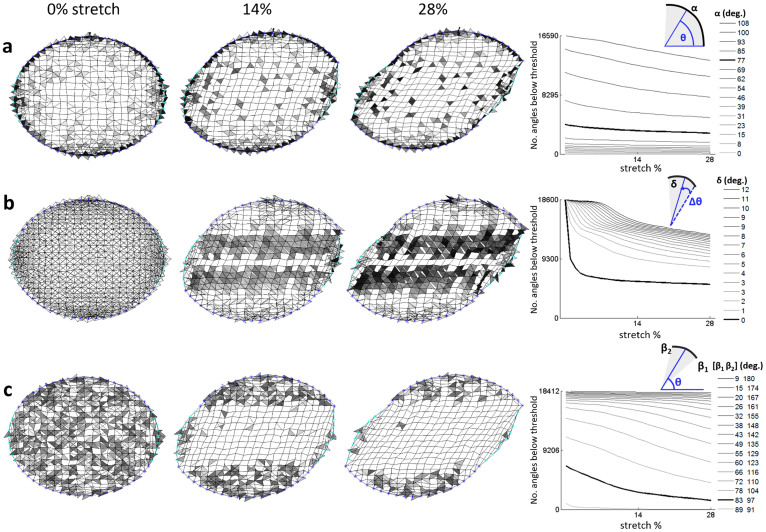
Mechanosensing thresholding models indicate distinct differences in crosslinker angle distributions that exceed fast-release threshold. Our model implements mechanosensing by linking structural changes in the actin network to biochemistry via FLNa-deformation-dependent FilGAP release. We examine three different mechanosensing models (a: absolute, b: delta, c: bandpass) dependent on crosslinking FLNa homodimer angle conformation (Fig. 1b). As the network stretches, shaded regions represent angles below threshold (i.e., to be released at slow rate) where the lighter the shade, the closer the angle is to the fast release threshold. Completely clear regions represent angles above threshold (i.e., to be released at fast rate). Representative square-grid actin filament networks from 0–28% stretch are shown using a (a) 77° absolute threshold, (b) 0° delta angle threshold (i.e.*,* any angle increase passes the threshold) and (c) 90 ± 7° range bandpass angle threshold; we illustrate these specific thresholds as they were optimized to experimental FilGAP release (Supplementary Fig. S5). The far right graphs show corresponding numbers of angles under threshold from 0-28% stretch for a range of thresholds. The bolded lines highlight the stated optimized threshold values. Simulation parameters: 421 intersections/internal nodes, 60 peripheral nodes, and 960 filaments averaged over 10 runs.

**Figure 4:**
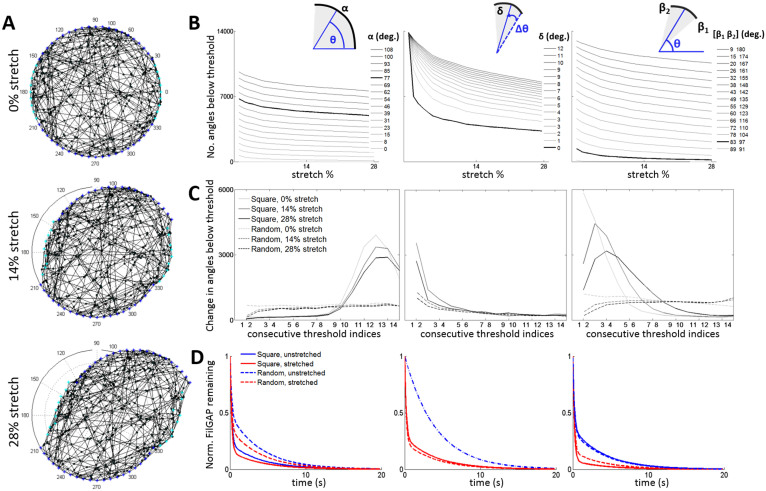
Disordered actin networks enable more finely tuned control of molecular release with a more robust response than do ordered square-grid networks. While maintaining the same parameters used in square-networks, we generate randomized actin network configurations and average results over 7 runs. (a) Representative random network under increasing stretch. (b) For absolute (left), delta (middle) and bandpass (right) thresholding, we plot the number of crosslink angles below threshold as stretch increases for a range of threshold values. The bolded lines highlight previously optimized thresholds using square networks (77° for absolute, 0° for delta and 90 ± 7° for bandpass). (c) For the thresholding models, we compare the marginal increase in number of angles below threshold for square vs. random networks as we increment consecutive thresholds. (d) Mixture model predictions for FilGAP release for square vs. random networks using the best fit threshold values.

The final parameters for the mixture model *N = A exp(−t/k_slow_) + B exp(−t/k_fast_) + C* fitted to the raw data are *k_slow_* = 4.0669 s, *k_fast_ = * 0.1876 s, and *C* = 0.0006. Whereas in our previous paper, it was assumed that a network consisted of a homogeneous population of crosslinks either releasing at fast or slow rates, our new mixture model takes into account that the networks consist of a combination of fast and slow releasing crosslinks. For a given actin network under a stretch, the two decays represent the network's time-dependent release of molecules such as FilGAP at both crosslinking sites favorable for slow release (angles under threshold) and angles favorable for fast release (angles exceeding threshold). The overall signal *N* represents the number of signaling molecules remaining in a specific network configuration as a function of time. The first exponential term describes slow decay: *A* represents the number of angles below threshold and *k_slow_*describes the rate of slow release. The second exponential describes fast decay: *B* describes the number of angles above threshold and *k_fast_* describes the rate of fast release; *C* represents background fluorescence noise. *A* and *B* are dependent on the number of angles below and above the fast release threshold, respectively, and are determined by geometric simulations of stretch on the actin network (Fig. 2).

## Author Contributions

J.K., P.R.L. and R.S.S. wrote the manuscript. The mechanotransductive model was designed by J.K., K.M.P., A.J.E., P.R.L. and R.S.S. A.J.E. provided experimental data. J.K. performed the computational simulations and analyzed the data. All authors contributed to the interpretation of the results and contributed to or commented on the manuscript.

## Supplementary Material

Supplementary Information Supplementary Figures and Tables

Supplementary Information Supplementary Movie S1: Ordered, square crosslink network, absolute threshold of 77°

Supplementary Information Supplementary Movie S2: Ordered, square crosslink network, delta threshold of 0°

Supplementary Information Supplementary Movie S3: Ordered, square crosslink network, bandpass threshold 90±7°

Supplementary Information Supplementary Movie S4: Disordered, random crosslink network, absolute threshold of 77°

Supplementary Information Supplementary Movie S5: Disordered, random crosslink network, delta threshold of 0°

Supplementary Information Supplementary Movie S6: Disordered, random crosslink network, bandpass threshold of 90±7°

## Figures and Tables

**Figure 3:**
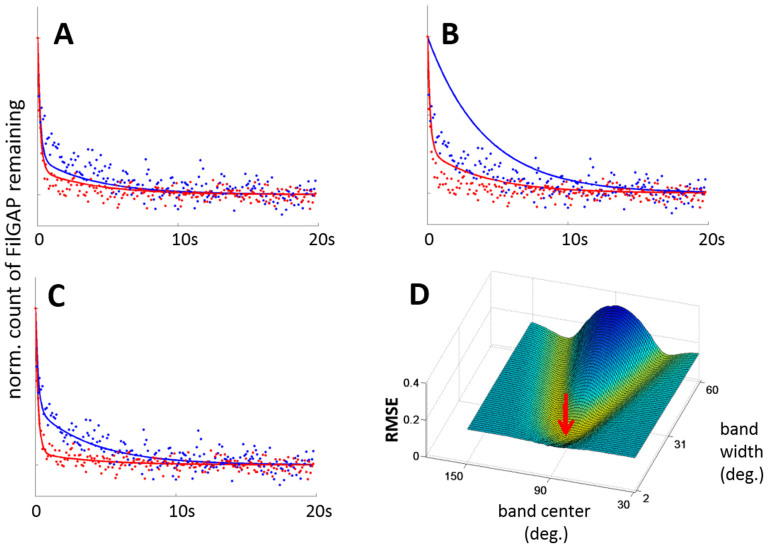
The bandpass threshold is optimal to model FLNa-FilGAP release under a square-grid network. We model mechanoresponse by simulating time-dependent release of FLNa-bound FilGAP as a mixture of slow- and fast-releasing populations (Equation 1). We compare the best fits from a parameter search (Supplementary Fig. S5) of (a) absolute, (b) delta and (c) bandpass thresholds as compared to fluorescent decay data of tagged FilGAP release from reconstituted actin networks^25^. 0% stretch data in blue and 28% stretch data in red. Best fits: (a) absolute: 77° threshold with RMSE 0.123, (b) delta: 0° threshold (i.e., any increase in angle) with RMSE 0.262, (c) bandpass: 90 ± 7° threshold with RMSE 0.100. (d) We perform a parameter search of both bandpass center (30 to 150°) and width (2 to 60°) and find that with two degrees of freedom, the optimal band is 92 ± 8° with a negligible RMSE improvement to 0.099 (red arrow). Simulation parameters: 421 intersections, 60 peripheral nodes, and 960 filaments averaged over 10 runs.
