# Supplementary material for: Structurally Governed Cell Mechanotransduction through Multiscale Modeling
Source: Sci Rep. 2015 Feb 27;5:8622. doi: 10.1038/srep08622 (PMC4342557; doi:10.1038/srep08622)
Supplement: Supplementary Information — Supplementary Figures and Tables [file srep08622-s1.pdf]

Structurally Governed Cell Mechanotransduction through Multiscale Modeling

John Kang[1], Kathleen M. Puskar[2], Allen J. Ehrlicher[3], Philip R. LeDuc[1,4], Russell S. Schwartz[1,5*]

Affiliations:

[1]Lane Center for Computational Biology, Carnegie Mellon University, Pittsburgh, PA 15213, USA.

[2] Dept. of Mechanical Engineering Technology, Point Park University, Pittsburgh, PA 15222, USA.

[3]Dept. of Bioengineering, McGill University, Montreal, Quebec H3A 0C3, Canada.

[4]Dept. of Mechanical Engineering, Carnegie Mellon University, Pittsburgh, PA 15213, USA.

[5]Dept. of Biological Sciences, Carnegie Mellon University, Pittsburgh, PA 15213, USA.

*Corresponding author. E-mail: russells@andrew.cmu.edu

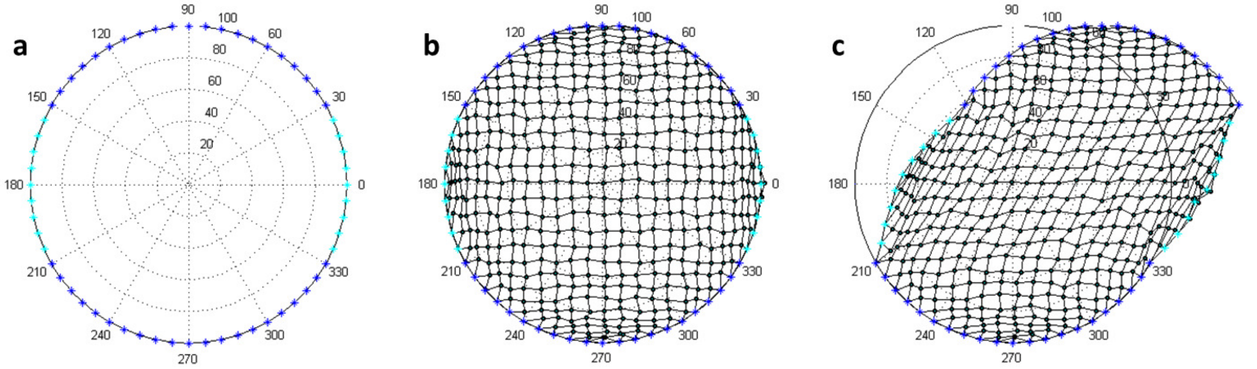

Supplementary Figure S1: Ordered square-crosslink actin network generation and stretching. **a**, fixed perimeter nodes (blue) and mobile perimeter nodes (cyan) are generated along the periphery. **b**, opposing perimeter nodes are connected to form filaments and the intersecting crosslinks are represented as black dots. 3% noise in X-Y coordinates is introduced to each crosslink. Each intersection represents a molecular complex containing four potential binding sites at four angles. **c,** the apical fixed perimeter nodes have been displaced by 28% to the right (defined as the ratio of the displacement to the right over the height of the cell) causing strains on the mobile nodes (cyan and black). The stretched network reaches equilibrium after the mobile nodes iteratively relax their strains so that the sum of the strains on each node is minimized as previously described[1].

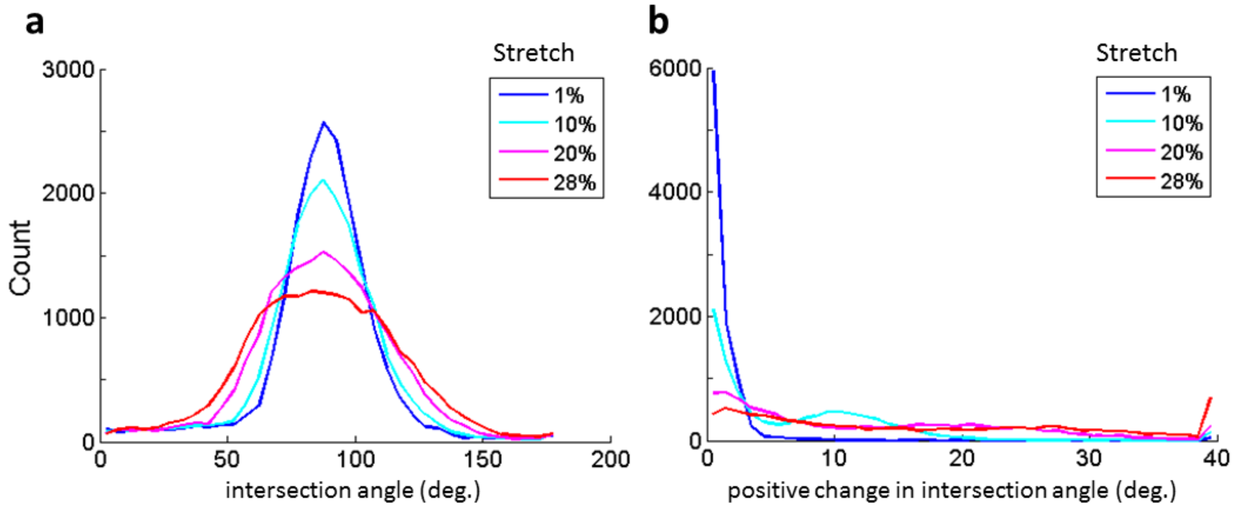

Supplementary Figure S2: Distribution of (**a**) the intersection crosslinking angles and (**b**) positive increases in intersection angles (i.e., delta angles) in an ordered square-crosslink actin network model. Results shown averaged over 10 runs. Model parameters: 421 internal nodes, 60 peripheral nodes, and 960 filaments (**Fig. 2**).

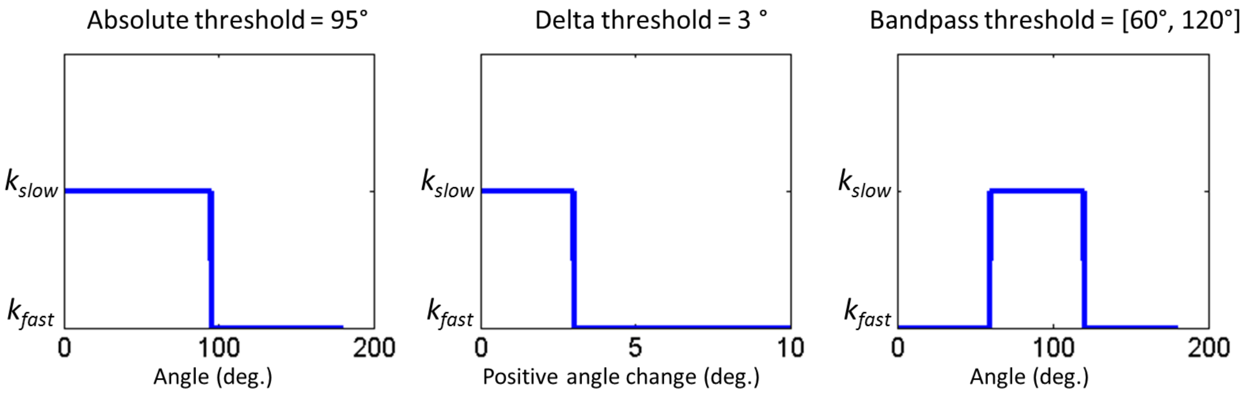

Supplementary Figure S3: Examples of release rate response vs. angle value for three thresholding models (absolute, delta and bandpass). These thresholds determine the rate of release of a molecule at a crosslinking angle. (Left) absolute angle thresholding with threshold of 95 degrees, (Middle) delta angle thresholding with threshold of 3 degrees, (Right) bandpass angle thresholding with threshold band of [60, 120] degrees. For further details of the thresholding models, see **Fig. 1b**.

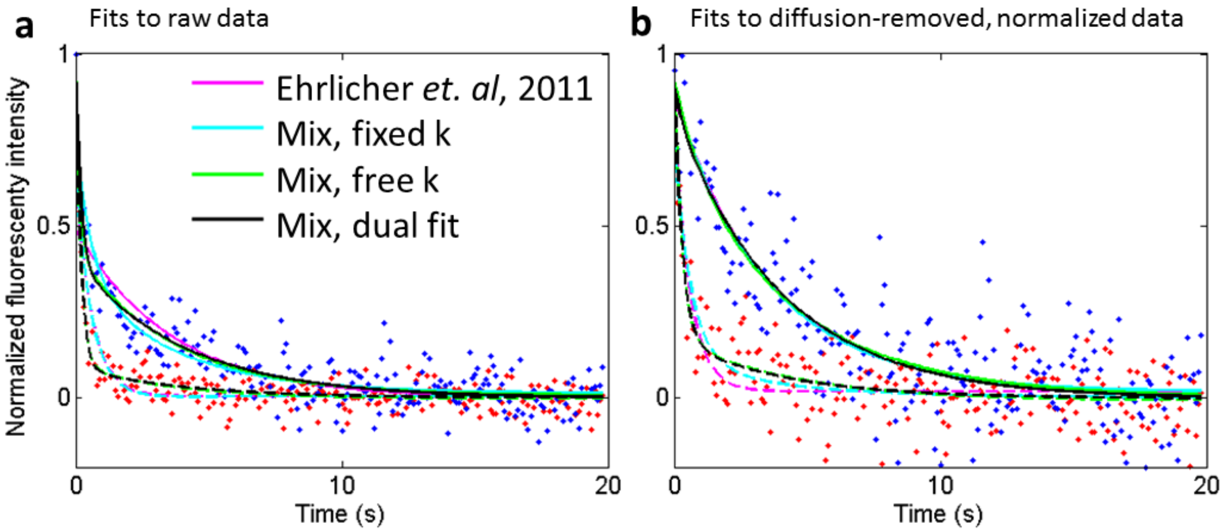

c **Raw data**

0% stretch	RMSE	r^2
Ehrlicher	0.0725	0.73
Fixed K	0.0636	0.793
Free K	0.06	0.815
Dual Fit	0.0602	0.815

28% stretch	RMSE	r^2
Ehrlicher	0.0517	0.717
Fixed K	0.0517	0.717
Free K	0.0385	0.843
Dual Fit	0.0387	0.843

d **Diffusion-removed, normalized data**

0% stretch	RMSE	r^2
Ehrlicher	0.124	0.74
Fixed K	0.124	0.74
Free K	0.124	0.742
Dual Fit	0.124	0.74

28% stretch	RMSE	r^2
Ehrlicher	0.0891	0.563
Fixed K	0.0869	0.585
Free K	0.0836	0.616
Dual Fit	0.0837	0.615

Supplementary Figure S4: Fits of three alternative mechanoresponse models and our chosen model ("Mixture model, dual fit"). Figures demonstrating fits to (**a**) raw and (**b**) diffusion-removed data for comparison between the "Ehrlicher" (purple), "mix, fixed k" (cyan), "mix, free k" (green), and the "mix, dual fit"(black) models. Unstretched data in blue, stretched data in red. Solid lines denote fits to unstretched data, dashed lines to stretched data. See **Table S1** for the fitted parameters for each model. Tables demonstrating root mean squared error (RMSE) and squared correlation coefficients (r^2) for the models' best fits to (**c**) raw data and (**d**) diffusion-removed data. See **Supplementary Table S1** for full description of the models and diffusion normalization methodology.

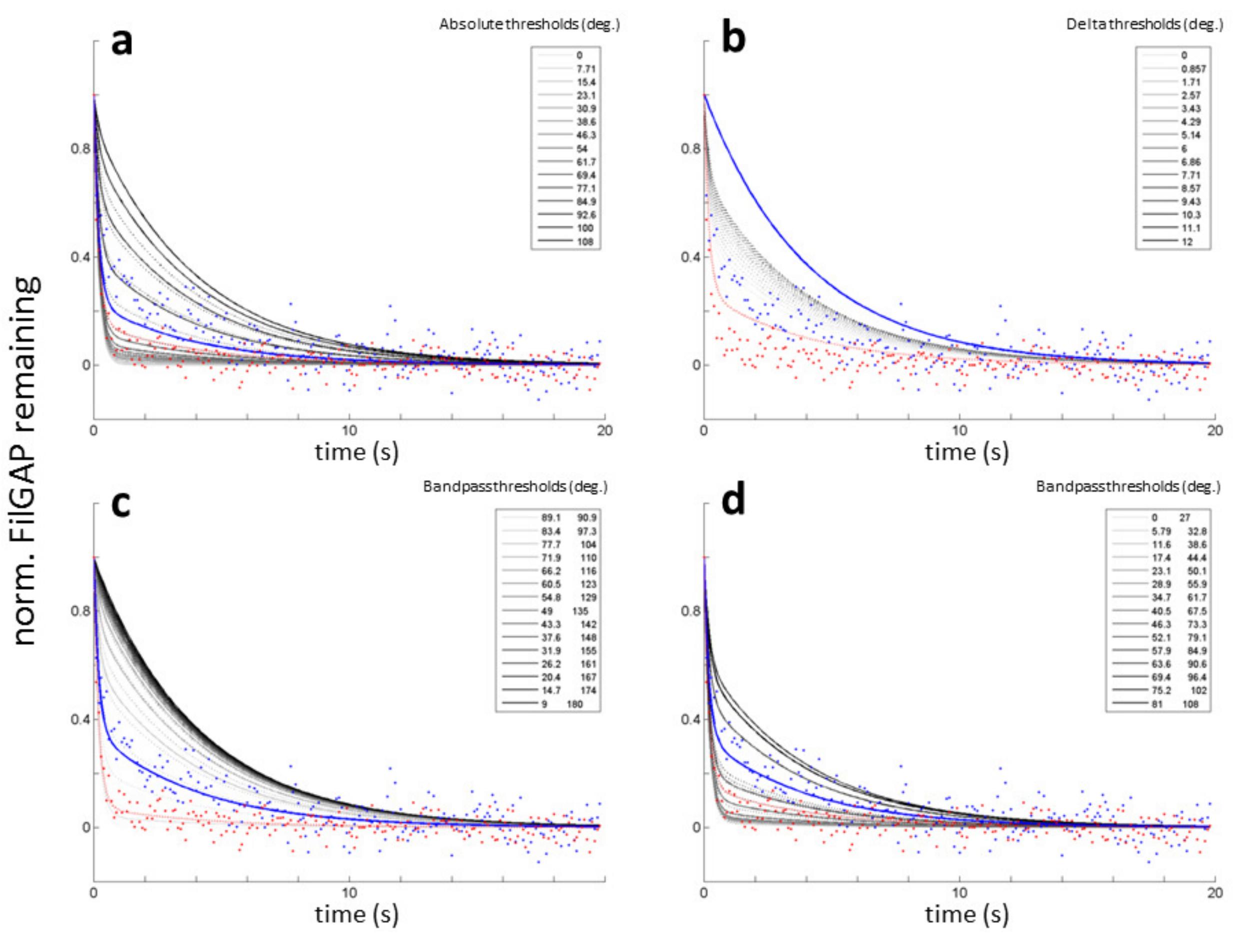

Supplementary Figure S5: Finding the optimal thresholds for the three thresholding mechanosensing models. We search a range of 15 threshold values for each model. Experimental data shown as red (28% stretched network) and blue (unstretched network). Best fit lines are colored in red (for stretched) and blue (for unstretched). (**a**) Absolute angle thresholds from 0° to 108°in 7.7° steps. Best fit at 77.1° with 0-10s RMSE of 0.145, 0-20s RMSE of 0.123. (**b**) Delta angle thresholds from 0° to 12°in 1° steps. Best fit at 0° with 0-10s RMSE of 0.356 and 0-20s RMSE of 0.262. (**c**) Bandpass angle thresholds with constant 90° center widened from [89.1°, 90.9°] to [9°, 180°] bands in 5.7° steps. Best fit at [83.4°, 97.3°] with 0-10s RMSE of 0.103 and 0-20s RMSE of 0.100. (**d**) Bandpass angle thresholds with constant width of 27° width shifted from [0°, 27°] to [81°, 108°] by 5.8° steps. Best fit at [57.9°, 84.9°] with RMSE=0.107.

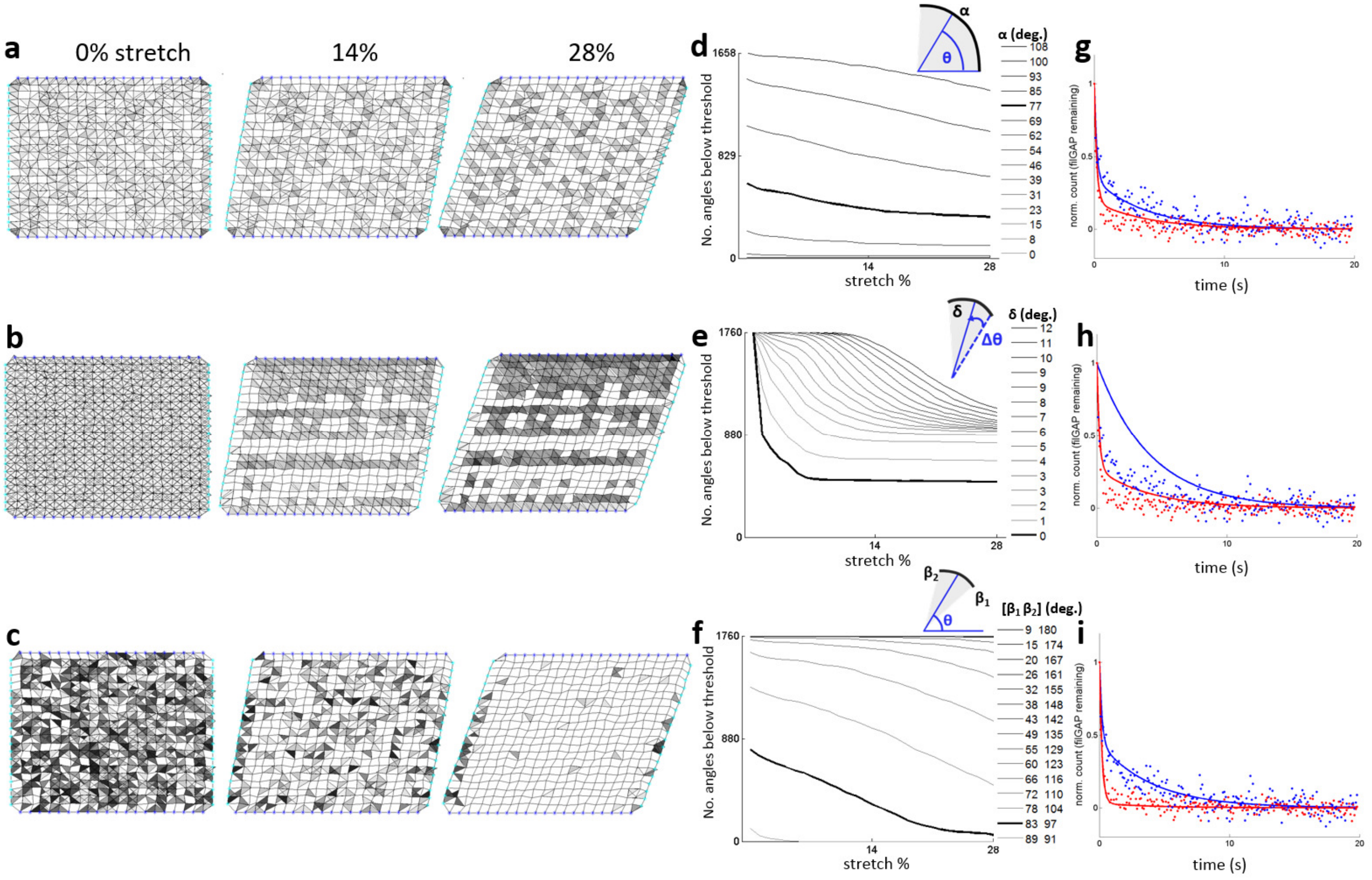

Supplementary Figure S6: Ordered square-perimeter square-crosslink actin network stress mechanosensing and mechanoresponse. Representative square-perimeter square-crosslink actin networks under 0-28% stretch are shown using a **(a)** 77° absolute threshold, **(b)** 0° delta angle threshold and **(c)** 90±7° bandpass angle threshold; these specific thresholds were found to fit best in our simulations to experimental FilGAP release. The square-perimeter geometry illustrated here more closely resembles the experimental flat plate setup[2]. Shaded regions represent angles below threshold (i.e., to be released at slow rate) where the lighter the shade, the closer the angle is to the fast release threshold. Completely clear regions represent angles above threshold (i.e., to be released at fast rate). Number of angles under threshold from 0-28% stretch for a range of thresholds are shown for **(d)** absolute, **(e)** delta and **(f)** bandpass thresholds. Bolded lines highlight the stated optimized thresholds. We compare the experimental vs. simulated time-dependent release of FilGAP for **(g)** absolute (RMSE 0.112), **(h)** delta (RMSE 0.259), and **(i)** bandpass thresholds (RMSE 0.104)[2]. Simulation parameters: 400 internal nodes, 80 peripheral nodes, and 920 filaments. 0% stretch data in blue and 28% stretch data in red.

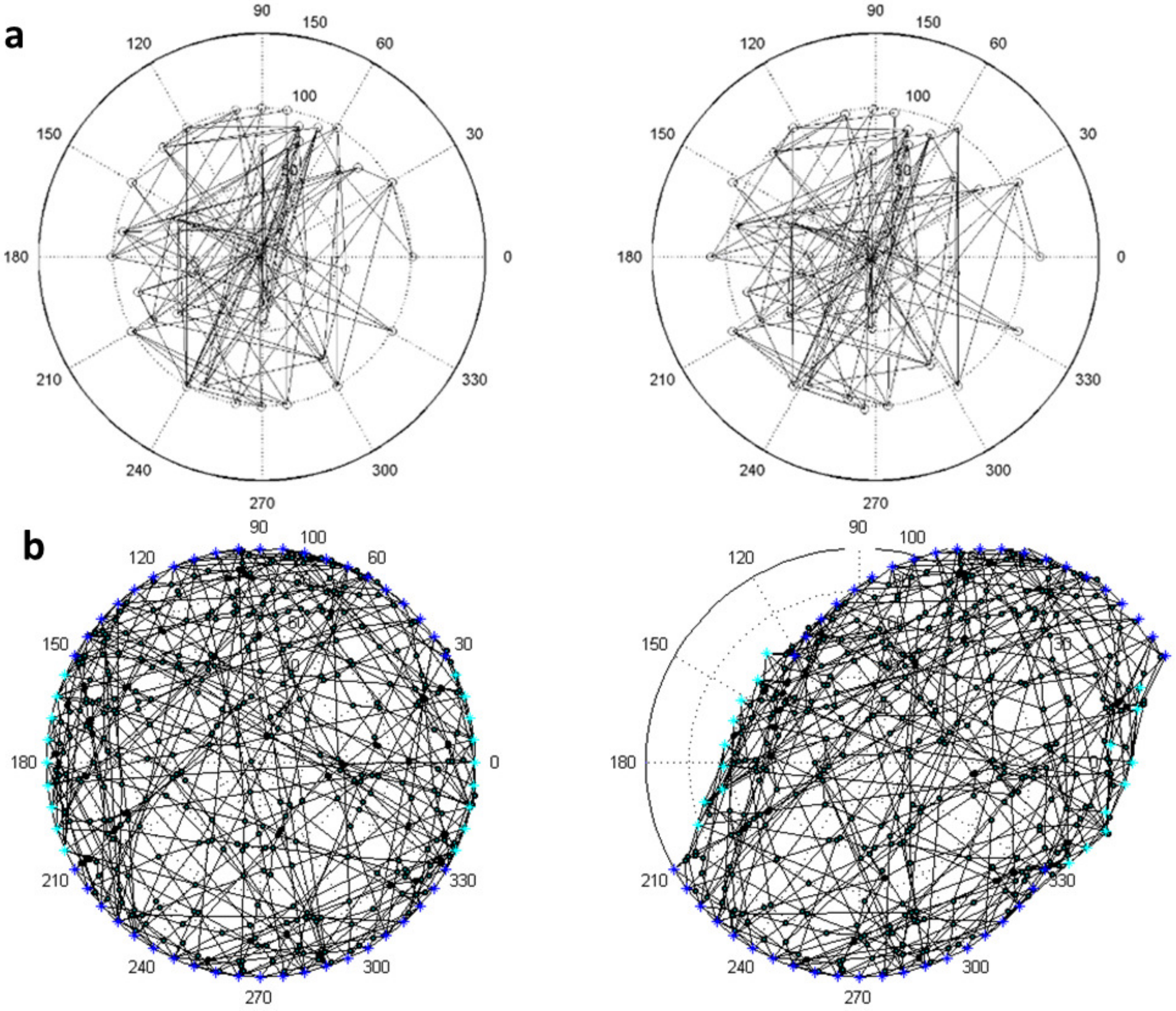

Supplementary Figure S7: Comparison of actin network models from this work and Kang *et al.* 2011. a, prior randomized actin network model used in our previous work to demonstrate actin filament alignment [1]. Left: 0% stretch, Right: 10% equatorial stretch. Note that certain internal nodes are connected by three or fewer filaments. **b**, current network model in a random configuration whereby each internal node (black dot) is stabilized by four filaments, symbolizing the crosslinking of actin filaments by filamin A. Left: 0% stretch, Right: 28% apical stretch.

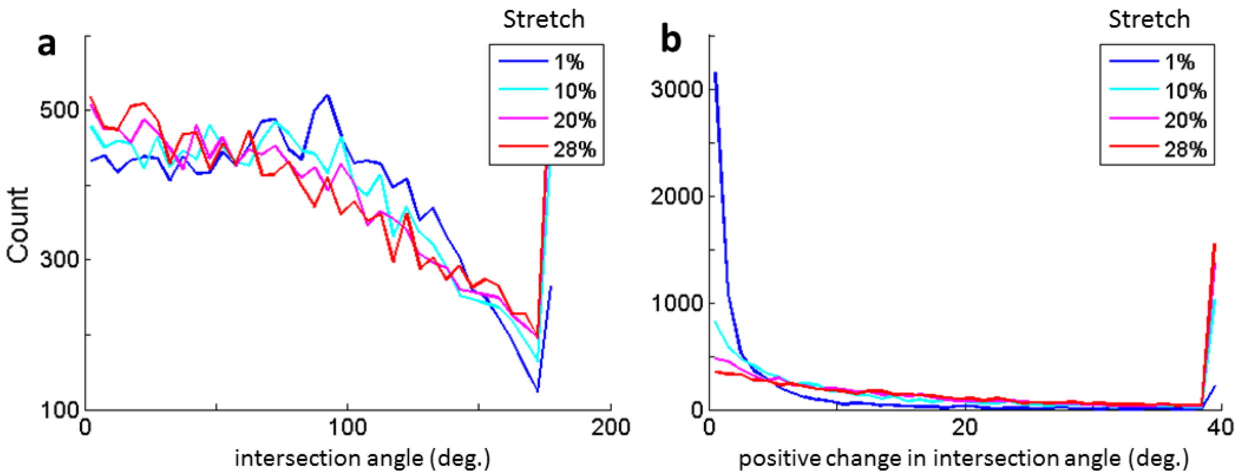

Supplementary Figure S8: Distribution of (**a**) intersection crosslinking angles and (**b**) positive increases in intersection angles (*i.e.,* delta angles) in a disordered random actin network model. Results shown averaged over 7 runs. Model parameters are 421 internal nodes, 60 peripheral nodes, and 960 filaments (see **Fig. 4**).

Supplementary Table S1: Fitting parameters to our chosen model ("mixture model, dual fit") and three tested alternatives. Curve fitting was performed to determine the constants k_{slow}, k_{fast}, and C for slow release rate, fast release rate and background noise, respectively. Here, we compare the parameters determined using alternative models to our final choice of a dual fit mixture model which uses one set of parameters to fit both 0% stretched and 28% stretched experimental data[2], which are denoted as $exp_0(t)$ and $exp_{28}(t)$, respectively. To also compare our parameters with our published results, we corrected for diffusion by subtracting $0.5e^{-t/0.15s}$ from the normalized raw data per Ehrlicher *et al.* [2] to generate diffusion-removed normalized data. Curve fitting was performed via the "nlinfit" MATLAB function using Levenberg-Marquardt nonlinear least squares algorithm for non-linear regression. The "Ehrlicher model, Nature 2011" model uses the single exponential fit that was performed in the eponymous paper[2] (4 parameters fit for 4 degrees of freedom, d.f.). The "Mixture model, fixed k's" model uses a two-exponential model whereby we assume the rate constants from Ehrlicher *et al.* describe the characteristic release rates for FilGAP (2 d.f.). The "Mixture model, free k's" model uses a two-exponential fit where all the parameters are freely floating such that a different set of parameters could be fit for unstretched *vs.* stretched data (6 d.f.). The "Mixture model, dual fit" model was the final model chosen for our analysis and also uses a two-exponential fit where the parameters must be constant between both unstretched and stretched data (3 d.f.). The final parameters taken from the raw data are $k_{slow} = 4.0669$s, $k_{fast} = 0.1876$s, and $C = 0.0006$. See **Supplementary Fig. S4** for plots and goodness-of-fit results.

Model	Objective function	d f	Raw data 0% stretch	Raw data 28% stretch	Corrected data 0% stretch	Corrected data 28% stretch
Ehrlicher model, Nature 2011	$exp_0(t) = A_0 e^{-\frac{t}{k_{slow}}} + C_0$<br>$exp_{28}(t) = A_{28} e^{-t/k_{fast}} + C_{28}$	4	$k_{slow} = 2.3011$<br>$C_0 = 0.0230$	$k_{fast} = 0.2521$<br>$C_{28} = 0.0121$	$k_{slow} = 3.6428$<br>$C_0 = 0.0151$	$k_{fast} = 0.5673$<br>$C_{28} = 0.0202$
Mixture model, fixed k's	$exp_0(t) = Ae^{-t/3.6} + Be^{-t/0.6} + C_0$<br>$exp_{28}(t) = Ae^{-t/3.6} + Be^{-t/0.6} + C_{28}$	2	$C_0 = 0.0140$	$C_{28} = 0.0067$	$C_0 = 0.0183$	$C_{28} = 0.0034$
Mixture model, free k's	$exp_0(t) = Ae^{-t/k_{slow,0}} + Be^{-t/k_{fast,0}} + C_0$<br>$exp_{28}(t) = Ae^{-t/k_{slow,28}} + Be^{-t/k_{fast,28}} + C_{28}$	6	$k_{slow,0} = 3.5197$<br>$k_{fast,0} = 0.1853$<br>$C_0 = -0.0017$	$k_{slow,28} = 3.6307$<br>$k_{fast,28} = 0.1437$<br>$C_{28} = 0.0074$	$k_{slow,0} = 4.4509$,<br>$k_{fast,0} = 1.1025$<br>$C_0 = 0.0015$	$k_{slow,28} = 4.3265$<br>$k_{fast,28} = 0.2777$<br>$C_{28} = -0.0078$
Mixture model, dual fit	$\left(exp_0(t) - A_0 e^{-\frac{t}{k_{slow}}} - B_0 e^{-\frac{t}{k_{fast}}} - C\right)^2 +$<br>$\left(exp_{28}(t) - A_{28} e^{-\frac{t}{k_{slow}}} - B_{28} e^{-\frac{t}{k_{fast}}} - C\right)^2 = 0$	3	**Simultaneous fit with raw 0% and 28% stretched data:**<br>$k_{slow} = 4.0669$, $k_{fast} = 0.1876$<br>$C = 6.1196e\text{-}04$		**Simultaneous fit with corrected 0% and 28% stretched data:**<br>$k_{slow} = 3.9804$, $k_{fast} = 0.2908$<br>$C = -5.4411e\text{-}004$	

Supplementary Table S2: Optimal threshold values show some sensitivity to changes in network density but order of best fitting threshold models is robust. Baseline parameters are altered to determine how the parameter density would affect overall results for our threshold models. Baseline parameters are 60 peripheral nodes (PN), 421 internal nodes (IN) and 960 filaments. A less dense network of 40 PN, 181 IN and 440 filaments and more dense network of 80 PN, 761 IN and 1680 filaments are compared. These two parameter sets were determined by altering the number of peripheral nodes which subsequently determine the number of intersections (internal nodes) and thus the filaments as well. Thresholds were incrementally tested using 1-2 degree steps from a range of 72-108 degrees for absolute, 0-12 degrees for delta and ±1-18 degrees around 90 degrees for bandpass. For all densities tested, bandpass threshold was the favored model determined by RMSE fitting to raw data, followed by absolute and lastly delta thresholds.

	**Baseline**	**Less dense**	**More dense**
**Peripheral node no.**	60	40	80
**Internal node no.**	421	181	761
**Filaments no.**	960	440	1680
**Best absolute (RMSE)**	77° (0.123)	82° (0.125)	77° (0.117)
**Best delta (RMSE)**	0° (0.262)	0° (0.275)	0° (0.248)
**Best bandpass (RMSE)**	90 ± 7° (0.099)	90 ± 6° (0.100)	90 ± 9° (0.099)

Supplementary Movies: visualizing angles below threshold for different network configurations and thresholding models

For all movies, 0-28% stretch is shown at 1% intervals. Shaded regions represent angles below threshold (i.e., to be released at the slow rate) where the lighter the shade, the closer the angle is to the fast release threshold. Completely clear regions represent angles above the threshold (i.e., to be released at the fast rate). Simulation parameters used were 421 internal nodes, 60 peripheral nodes, and 960 filaments.

Supplementary Movie S1: Ordered, square crosslink network, absolute threshold of 77°

Supplementary Movie S2: Ordered, square crosslink network, delta threshold of 0°

Supplementary Movie S3: Ordered, square crosslink network, bandpass threshold 90±7°

Supplementary Movie S4: Disordered, random crosslink network, absolute threshold of 77°

Supplementary Movie S5: Disordered, random crosslink network, delta threshold of 0°

Supplementary Movie S6: Disordered, random crosslink network, bandpass threshold of 90±7°

References

1 Kang, J. *et al.* Response of an actin filament network model under cyclic stretching through a coarse grained Monte Carlo approach. *J. Theor. Biol.* **274**, 109-119, doi:10.1016/j.jtbi.2011.01.011 (2011).

2 Ehrlicher, A. J., Nakamura, F., Hartwig, J. H., Weitz, D. A. & Stossel, T. P. Mechanical strain in actin networks regulates FilGAP and integrin binding to filamin A. *Nature* **478**, 260-263, doi:10.1038/nature10430 (2011).
