# Supplementary figures and images for: Structurally Governed Cell Mechanotransduction through Multiscale Modeling
Source: Sci Rep. 2015 Feb 27;5:8622. doi: 10.1038/srep08622 (PMC4342557; doi:10.1038/srep08622)

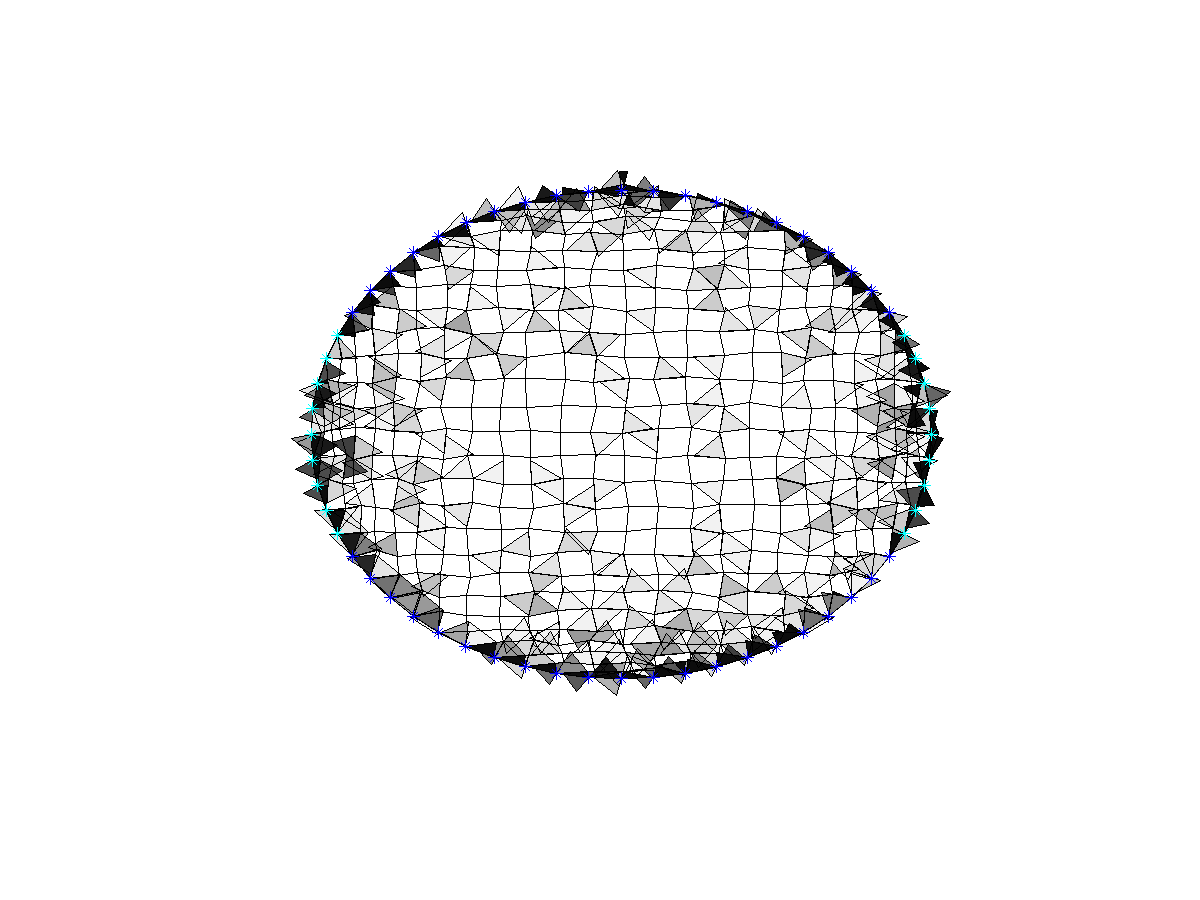

Supplement: Supplementary Information — Supplementary Movie S1: Ordered, square crosslink network, absolute threshold of 77° [file srep08622-s2.gif]

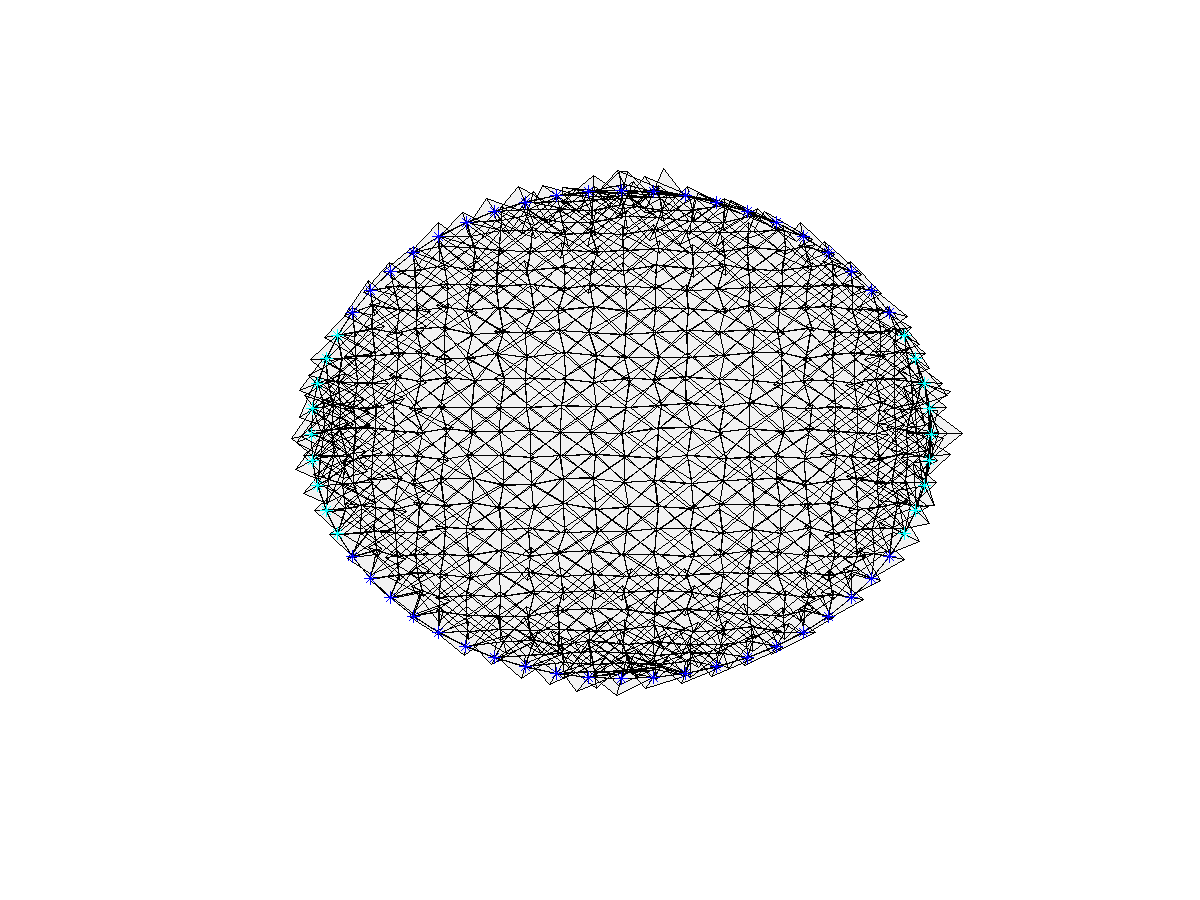

Supplement: Supplementary Information — Supplementary Movie S2: Ordered, square crosslink network, delta threshold of 0° [file srep08622-s3.gif]

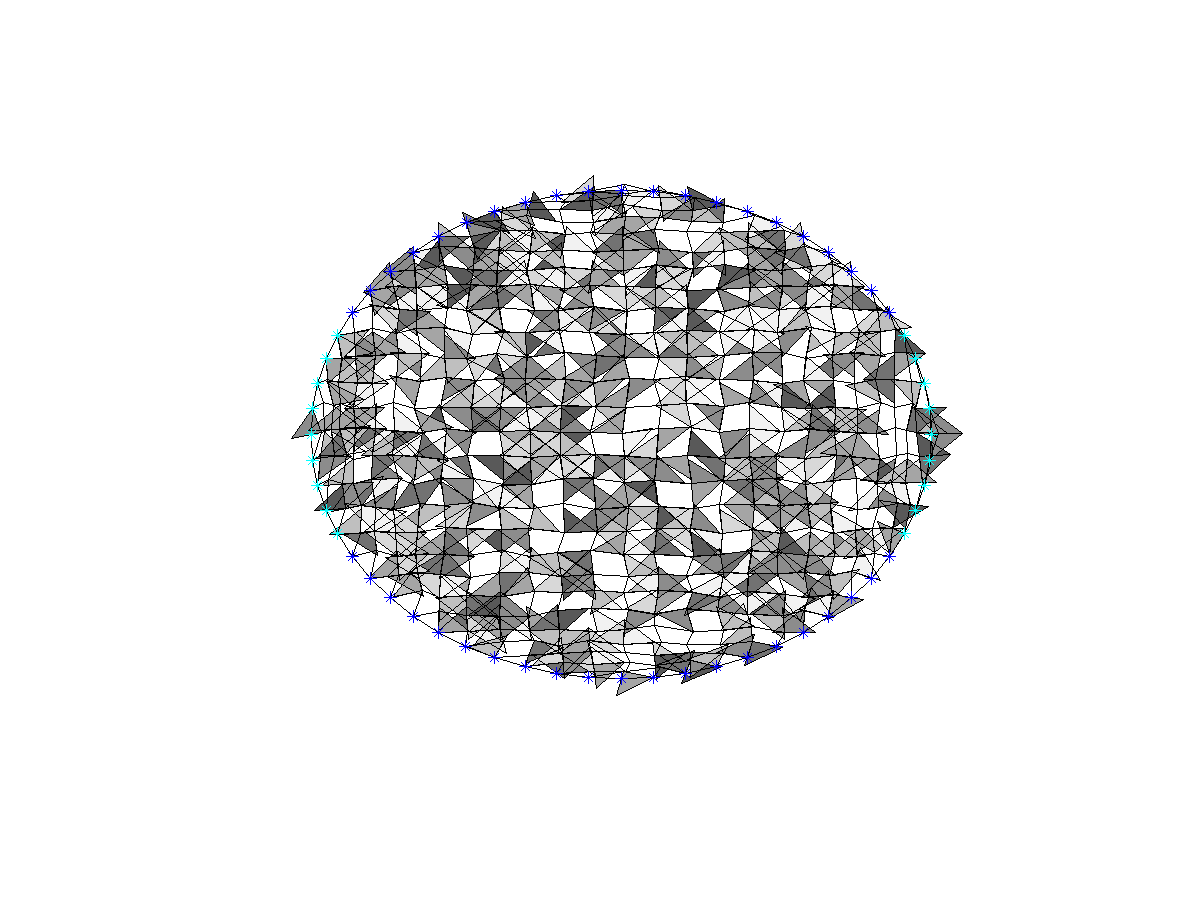

Supplement: Supplementary Information — Supplementary Movie S3: Ordered, square crosslink network, bandpass threshold 90±7° [file srep08622-s4.gif]

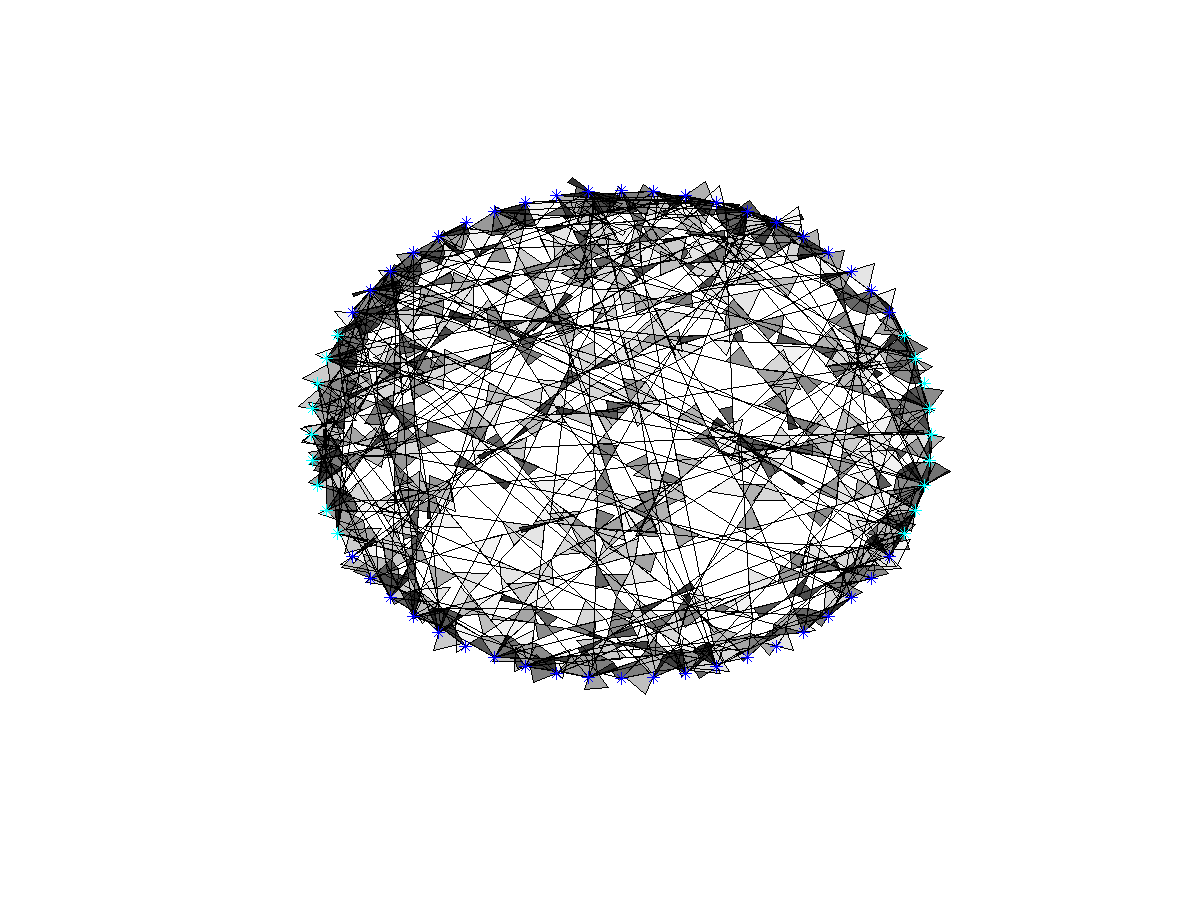

Supplement: Supplementary Information — Supplementary Movie S4: Disordered, random crosslink network, absolute threshold of 77° [file srep08622-s5.gif]

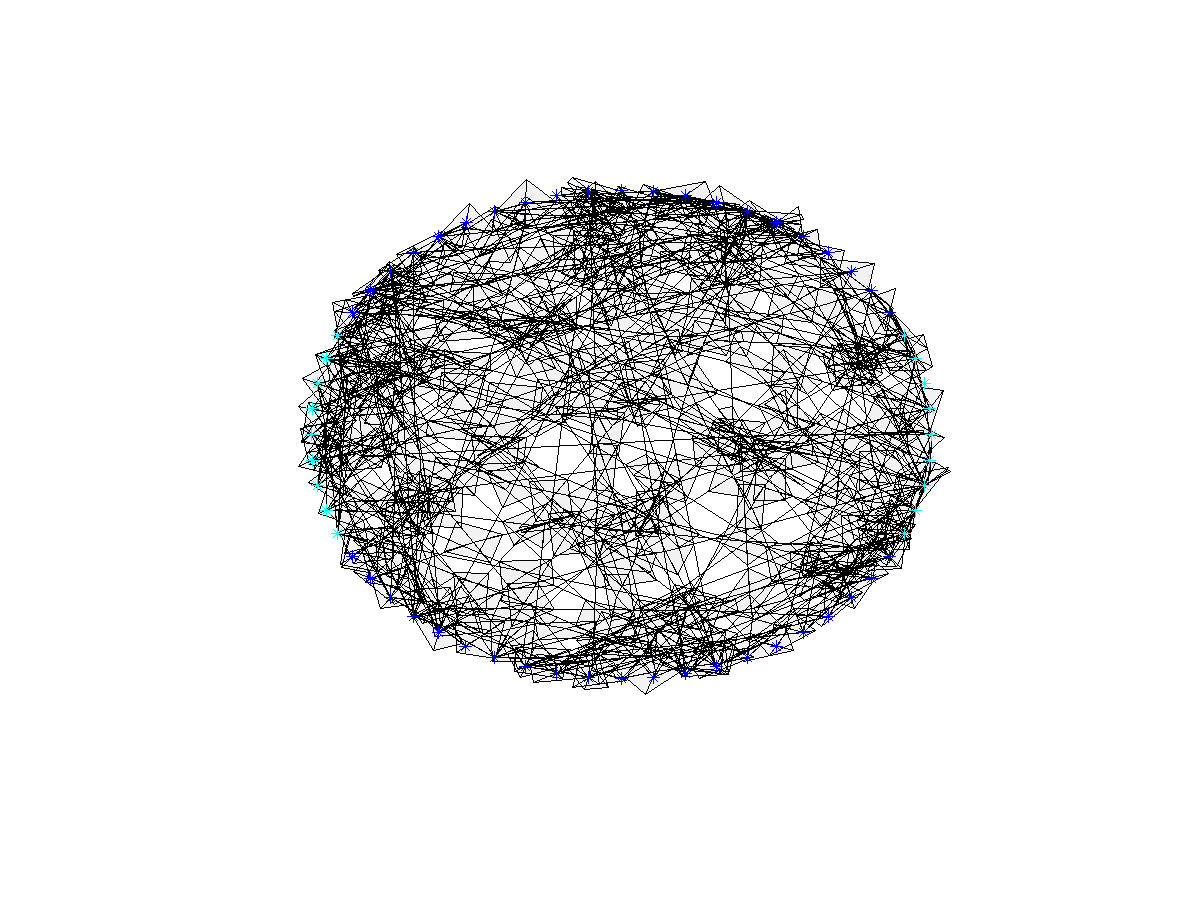

Supplement: Supplementary Information — Supplementary Movie S5: Disordered, random crosslink network, delta threshold of 0° [file srep08622-s6.gif]

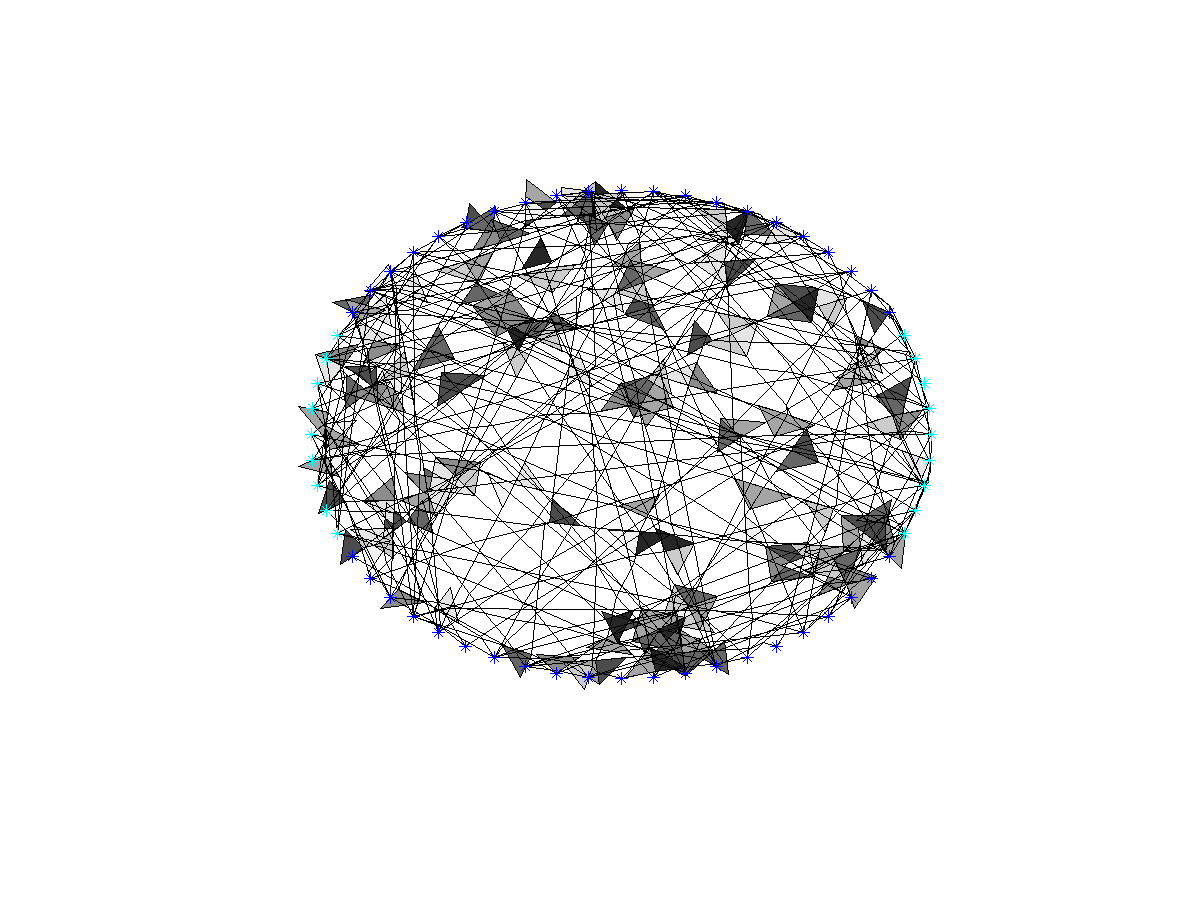

Supplement: Supplementary Information — Supplementary Movie S6: Disordered, random crosslink network, bandpass threshold of 90±7° [file srep08622-s7.gif]
